# Accelerated Recovery: The Role of Oral Steroid in the Management of Herpes Zoster Ophthalmicus-Related Ophthalmoplegia

**DOI:** 10.7759/cureus.50553

**Published:** 2023-12-15

**Authors:** Joobin Khadamy, Pardis Khademi, Sadjad Baharypour

**Affiliations:** 1 Ophthalmology, University Hospital of Umeå, Umeå, SWE; 2 Ophthalmology, St. Erik Eye Hospital, Stockholm, SWE; 3 Internal Medicine, Hospital Marienstift, Braunschweig, DEU

**Keywords:** third nerve palsy, oculomotor nerve palsy (omp), third cranial nerve (oculomotor nerve) palsy, corticosteroid treatment, herpes zoster ophthalmicus (hzo), herpes zoster ophthalmicus-related ophthalmoplegia (hzoro)

## Abstract

The use of systemic steroids in managing herpes zoster ophthalmicus-related ophthalmoplegia (HZORO) remains a topic of debate. Here, a case involving third nerve HZORO is highlighted, where a regimen of oral valacyclovir followed by a brief course of oral steroids resulted in significant improvement within days and complete resolution of the palsy within a month of initiating the treatment. This case underscores the need for randomized controlled studies to definitively determine the efficacy of systemic steroids in alleviating or shortening the course of HZORO.

## Introduction

Herpes zoster ophthalmicus (HZO) showcases diverse manifestations, among which herpes zoster ophthalmicus-related ophthalmoplegia (HZORO) is a notable occurrence, affecting either the ipsilateral or contralateral eye. While studies have noted some grades of ophthalmoplegia in up to 31% of HZO cases, diplopia is relatively uncommon. HZORO primarily affects the third cranial nerve [1].

Despite the variability in presentations, ophthalmoplegia in otherwise healthy HZO cases often holds a favorable prognosis, with notable improvement typically observed over a few months. However, the effectiveness of antiviral treatment, with or without steroids, remains uncertain, lacking earlier randomized clinical trials [2-5]. In this reported case, a patient diagnosed with HZORO is detailed, outlining the management and course of the disease.

## Case presentation

**Figure 1:**
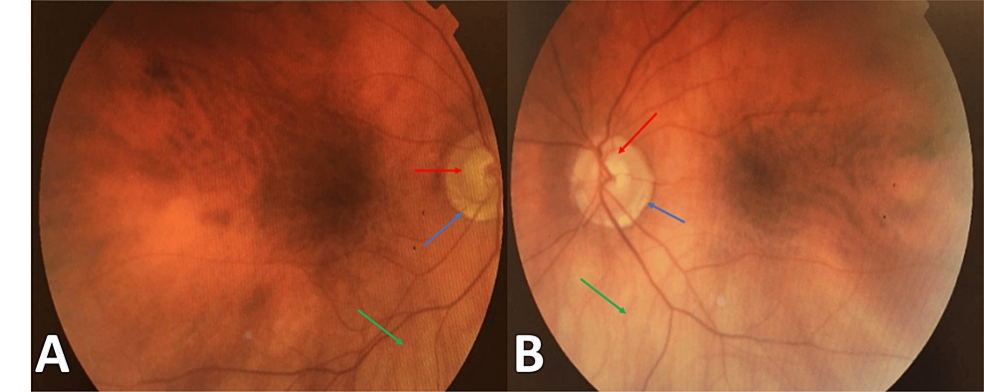
Fundus photo of the eyes. Images of the right (A) and left (B) eye's fundus reveal typical signs of myopic eyes. Noteworthy are the prominent choroidal vessels, indicating a thin retina (green arrow) and peripapillary atrophy (blue arrow). Additionally, signs of glaucomatous changes are evident. These include a large excavation and a high cup-to-disc ratio in the right eye and a superior notch in the left eye (red arrow). However, no signs of optic disc swelling, vasculitis, retinitis, or vitritis are observed in either eye.

An 83-year-old man came to the emergency department, complaining of a painful and erythematous skin lesion on his forehead that had persisted for a week. Initially diagnosed with erysipelas, he began a regimen of clindamycin 150 mg twice a day. One day later, his pain intensified, and vesicular lesions appeared on his forehead and around his left eye. Consequently, he was referred to an ophthalmologist, who diagnosed him with HZO. Treatment commenced with oral valacyclovir 1 gram three times daily. Ocular examination revealed no abnormalities except for a myopic fundus and glaucomatous disc, with no signs of vitreous or retinal vasculitis (Figure 1). Hutchinson's sign was not observed.

**Figure 2:**
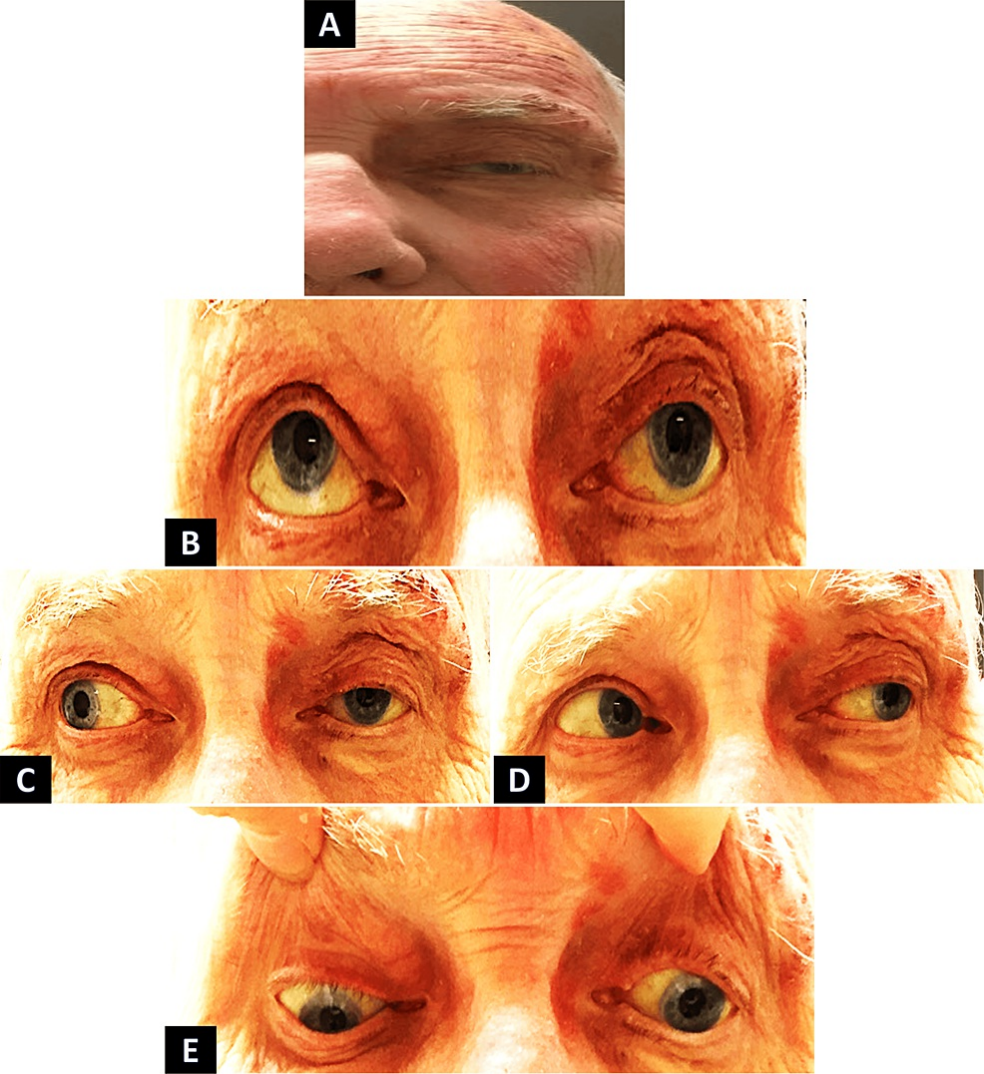
Third nerve HZORO in the left eye. HZORO: herpes zoster ophthalmicus-related ophthalmoplegia A: Visualizes crusted erythematous macules and papules in the distribution of the ophthalmic (V1) branch of the trigeminal nerve, along with ptosis and exotropia in the primary position. B: Illustrates a deficit in elevation and mild ptosis in the left eye. C: Demonstrates limited adduction in the left eye during right gaze. D: Displays normal abduction in the left eye during left gaze. E: Depicts a deficit in depression during downward gaze in the left eye.

The patient was taking bimatoprost 0.03%/timolol 0.5% ophthalmic solution (Ganfort, Allergan, Dublin, Ireland). A day later, he returned to the clinic, reporting diplopia. Examination indicated restricted adduction, elevation, and depression, coupled with ptosis of the left eyelid (Figure 2). Corneal sensation was normal. The pupil responded symmetrically and reactively, and no relative afferent pupillary defect (RAPD) was detected.

Orbital magnetic resonance imaging (MRI) results were normal, ruling out myositis and orbital apex syndrome. Brain magnetic resonance angiography (MRA) and venography (MRV) were performed to rule out vascular involvement or cavernous sinus issues, revealing only white substance degenerative changes indicating old right parietal lobe infarcts.

**Figure 3:**
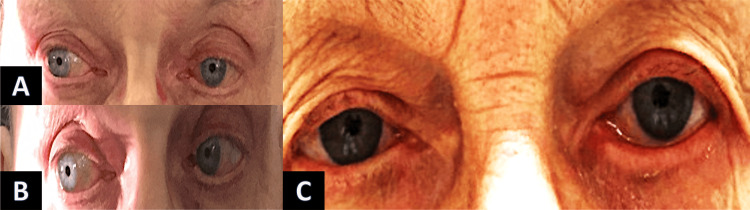
Improvement of eye motility during follow-up. A: By the third day of steroid treatment initiation, the patient showed diminished restrictions in eye movements, and subjective diplopia in the primary position lessened. B and C: Without additional therapy, after a month, the paralysis fully resolved, and the patient achieved orthotropia in the primary position.

Additionally, 70 mg/day of oral prednisolone (1 mg per kg) was initiated for three days and tapered over seven days. Within three days of starting the steroid, the patient displayed reduced limitations in eye movements, and subjective diplopia in the primary position decreased. After one month without any further therapy, the palsy completely resolved (Figure 3).

## Discussion

In HZORO, while approximately 90% of cases manifest with rashes, less than 10% exhibit diplopia [3]. The sites affected causing motility impairments encompass muscles and neurons across multiple levels, including the orbital apex, cavernous sinus, and central nervous system [1]. These impairments result from various mechanisms such as viral cytopathic effects, immune responses, and vasculitis [1,3]. Our case presented with ipsilateral pupil-sparing oculomotor palsy, which occurred despite the initiation of antiviral treatment.

Notably, prior reports demonstrated comparable recovery rates between oral and intravenous antiviral therapies, irrespective of treatment duration surpassing 10 days or being 10 days or less [3]. The role of corticosteroids in treating HZORO remains controversial. A recent systemic review highlighted a more favorable prognosis for women, immunocompetent individuals, and those administered corticosteroids [3]. Another recently published review article showed that the complete recovery rate among immunocompetent patients with HZORO remained consistent between individuals treated solely with antivirals and those receiving a combination of antivirals and oral steroids. Nonetheless, it was noted that age might play a role in the recovery of ophthalmoplegia [2]. Additionally, a recent meta-analysis indicated that prolonged steroid therapy yielded positive effects, offering a potential avenue for improving recovery from ophthalmoplegia associated with HZO, while age, gender, and initial steroid dosage didn't notably impact recovery status [4]. These results emphasize the potential advantages of investigating extended steroid tapering as a feasible strategy in managing HZORO, urging the need for further explorations.

A prior study showed that fewer than 40% of patients experienced complete recovery, leaving over 60% with persistent ophthalmoplegia. Typically, substantial improvement was noted within two months [5]. Our patient demonstrated a swift and full recovery within a month of commencing a 10-day regimen involving both antiviral and corticosteroid treatments. 

## Conclusions

The management of HZORO remains a challenge, particularly regarding the use of systemic steroids. This case contributes to the scarce evidence suggesting that corticosteroids might significantly improve recovery in HZORO cases. The highlighted instance of third nerve HZORO demonstrated marked improvement within days and complete resolution of the palsy within a month with a short course of oral valacyclovir and steroids. This emphasizes the critical need for randomized controlled trials to conclusively ascertain the efficacy of systemic steroids in easing or abbreviating the course of HZORO. Furthermore, determining the optimal dosage and duration of corticosteroid treatment is imperative for refining HZORO management strategies.
